# Hydrogels and Their Functionalization—Analysis of the Possibility of Their Application in Post-Fire Water Treatment Processes

**DOI:** 10.3390/ma17235820

**Published:** 2024-11-27

**Authors:** Anna Rabajczyk, Justyna Gniazdowska, Piotr Stojek, Katarzyna Czyżewska, Anna Trusek, Karolina Labus

**Affiliations:** 1 Scientific and Research Centre for Fire Protection—National Research Institute, Aleja Nadwiślańska 213, 05-420 Józefów, Poland; jgniazdowska@cnbop.pl (J.G.); pstojek@cnbop.pl (P.S.); 2 Department of Micro, Nano and Bioprocess Engineering, Faculty of Chemistry, Wrocław University of Science and Technology, 50-373 Wrocław, Poland; katarzyna.czyzewska@pwr.edu.pl (K.C.); anna.trusek@pwr.edu.pl (A.T.); karolina.labus@pwr.edu.pl (K.L.)

**Keywords:** fire wastewater, hydrogels, modification, water purification, environmental safety

## Abstract

Increasingly intense changes in climatic conditions and the use of modified materials are causing fires, the consequences of which are increasingly serious for the environment. On one hand, there is the issue of access to water resources. On the other hand, there is the problem of post-fire wastewater, which often contains a mixture of simple inorganic compounds and complex organic molecules, making the removal of pollutants a difficult task requiring innovative approaches. Among these solutions, hydrogels stand out as a promising class of sorption materials. Depending on their synthesis or functionalization, hydrogels can effectively capture contaminants and facilitate the reduction or removal of specific pollutants. This study explores the functionalization of polymeric materials, specifically hydrogels, using microorganisms or bioactive substances to create materials capable of treating water contaminated with hazardous substances generated during firefighting incidents. The possibility of wastewater capture was also taken into account to retain pretreated water at the place of pollutant generation. The analysis covered the potential, conditions, and limitations of using hydrogels in post-fire operations for the effective management of contaminated waters. It was shown that hydrogels, depending on the modification, have the potential to capture wastewater and purify it from both organic and inorganic substances specific to post-fire wastewater. However, it is not possible for a given hydrogel to meet all desired expectations at the same time. Furthermore, modifications that facilitate the optimal performance of certain functionalities may render the others ineffective.

## 1. Introduction

Deterioration of surface and groundwater quality is primarily due to expansive human activities. Processes that significantly affect the quality of these waters are mining, metallurgy, animal husbandry, the production and disposal of industrial, municipal, and agricultural waste, or soil erosion caused by land use changes [1]. Other factors, including natural phenomena such as precipitation or hydrological droughts, also contribute to the changes. Physicochemical properties are therefore the product of many factors that characterize a catchment area. However, water quality is increasingly affected, among other things, by major accident events and also fires [2,3]. Urbanization and more and more fires, covering larger and larger areas, are contributing to the release of considerable numbers of diverse pollutants. Nevertheless, not only the products of combustion of various substances and materials are emitted into the environment, but also the chemical compounds used during rescue operations, including fire extinguishing agents (e.g., foam-forming substances, fire extinguishing powders) or substances resulting from chemical reactions occurring in fire extinguishing agents during an incident [3,4]. Thus, post-fire waters generated from rescue and firefighting operations contain agents used during the action, such as solutions of foam extinguishing agents, waters used to neutralize chemicals, clean up accident sites, or water from water curtains, which further contributes to the complex matrix. Improper handling of such a diverse chemical mixture can cause short- and long-term negative environmental effects [4].

Fire extinguishing agents used in rescue and firefighting operations have both inorganic and organic substances in their composition. Among inorganic compounds, we can mention NaHCO_3_, KHCO_3_, NH_2_CONH_2_, Na_2_B_4_O_7_·10H_2_O, KCl, and NaCl, which in small amounts can be neutral to aquatic ecosystems. However, in higher concentrations, located in one place, they can threaten the balance of surface water. In the case of organic compounds, which are the primary components of foam extinguishing agents used in firefighting actions, their derivatives, such as anionic alkyl sulphates, amphoteric fluorinated alkyl amides, and anionic fluorinated alkyl sulphonates, are also present. Extinguishing agents can also contain nanoparticles, such as ZrO_2_ or NiO, iron(V) pentacarbonyl Fe(CO)_5_, bromotrifluoromethane CF_3_Br, or perfluorinated alkylated substances (PFASs), including perfluorooctanoic acid (PFOA) [5]. In case of PFOA, in accordance with the European Union Commission Regulation 2017/1000 of 13 June 2017, amending Annex XVII to Regulation (EC) No. 1907/2006 of the European Parliament and of the Council concerning the Registration, Evaluation, Authorization, and Restriction of Chemicals (REACH) with regard to perfluorooctanoic acid (PFOA), its salts, and derivatives [6], as of 4 July 2020, foam-forming agents containing perfluorooctanoic acid (PFOA) or its salts in concentrations equal to or greater than 25 ppb of PFOA, including its salts, or 1000 ppb of one derivative substance or a combination thereof, may not be manufactured or placed on the market. However, it should be noted that the new regulation does not eliminate the environmental problem, as the compound is currently being replaced by its homologs. In this case, the most commonly used is perfluorohexanoic acid (PFHxA), which, similar to other perfluoroalkyl substances (PFAS), is also a persistent organic chemical compound and suspected carcinogen. Therefore, solutions are being introduced based on the use of other surfactants that do not contain fluorine, which involves replacing the agents currently used in the installations. However, such replacements are costly and include the cost of redesigning, recalibrating, and cleaning existing systems due to changes in the physicochemical parameters of fluorosurfactant-free agents compared to concentrates containing fluorosurfactants, resulting primarily from the difference in viscosity and density of these types of agents, which forces significant changes in dosing systems. It also involves the need to dispose of agents that, once removed from the plant, are hazardous waste that is difficult to process and dispose of [7]. Thus, as a result of rescue and firefighting operations, depending on the extinguishing agent used and the materials and elements involved in the fire, wastewater is generated, the physicochemical parameters of which vary greatly (Table 1).

Substances released during fire and firefighting operations can enter directly into the environment or, in the case of an incident occurring in a developed area, into the sewage or combined sewerage system. This can contribute to an increase in the contamination of the soil and aquatic environment, including, among other things, disruption of the fish respiratory system, buffer imbalance, and increased risk of eutrophication [12]. It should be noted that the increase in pollution affects drinking water intakes as well as fishing sites, water bathing areas, or irrigation of agricultural fields with surface water, which can also indirectly affect the economy or affect human health [4]. Water yield, sediment volume, and environmental temperature increase as a result of the fire. In some cases, the amount and type of contamination caused biological communities to disappear as a result of post-fire runoff, and the duration of post-fire effects in some individual cases was 15 years or longer [2,13].

Therefore, it can be concluded that fire water is one of the significant problems not only in Poland but also worldwide. An additional problem is that many wastewater treatment plants refuse to accept fire water, especially when it includes AFFF-type foaming fire extinguishing agents [2,5,14]. Fire wastewater may be diluted in such situations and then discharged into the industrial or municipal wastewater stream. However, the prerequisite is that the characteristics and volumetric contribution of the post-fire water are such that they do not interfere with the treatment process. In most cases, however, adequate prior treatment through various processes, including chemical, physico-chemical, or microbiological processes, is necessary [10].

The efficiency of treatment processes is enhanced at higher concentrations of pollutants removed [15]. This has led to the pursuit of various effective and cost-efficient methods for capturing wastewater and adequately pre-treating it on-site, or alternatively, concentrating it and subsequently pre-treating it, or even recovering certain substances. One possible solution to this problem is to use a matrix with a higher capture affinity for the compounds required for removal. In the case of liquid waste containing fire-extinguishing agents or petroleum hydrocarbons, this can be achieved through the use of hydrogels. This is made possible by the ability of some organic compounds and inorganic compounds to interact with polymeric matrices, allowing them to be extracted from the solution [16]. An additional advantage is the possibility of using hydrogels, which, in the case of positively charged polymers, such as chitosan, would allow interaction with anionic surfactants by a dipole-dipole mechanism [17]. Since the fluorosurfactants used in AFFF agents are strong organic acids, the use of such interactions should allow their efficient extraction from post-fire waters [15,17].

**Figure 1:**
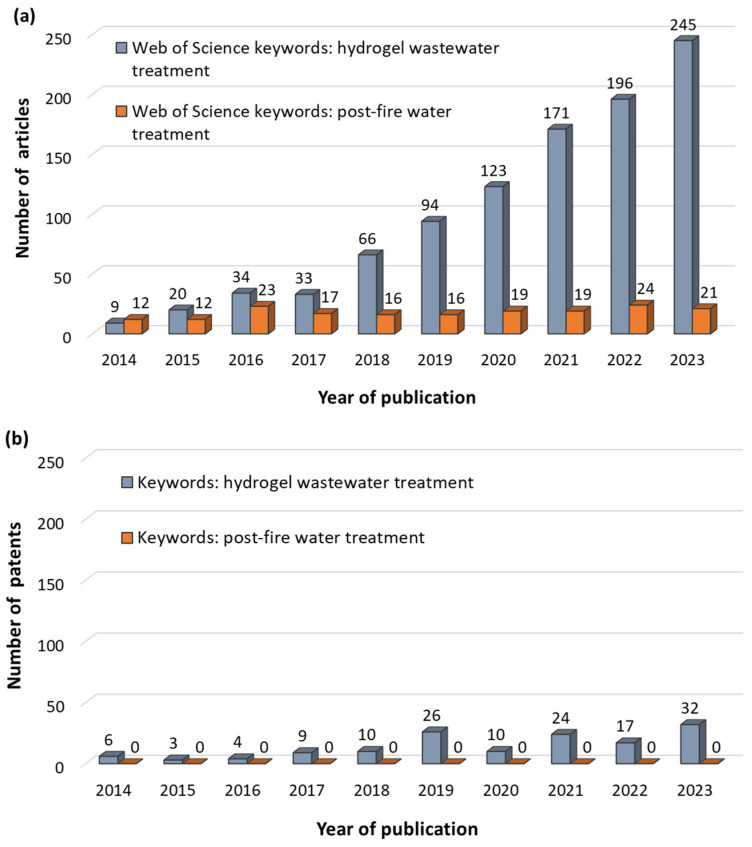
The number of articles (**a**) and patents (**b**) published from 2014 to 2023 analyzed for keywords: hydrogel wastewater treatment and post-fire water treatment in the search scope: *Topic—title, abstract, author keywords, and Keywords Plus*. Source: Web of Science database as well as patent bases such as Espacenet, GooglePatents, Patentscope, and European Patent Register (accessed on April 2024).

Therefore, the purpose of this article is to analyze the possibilities of using hydrogels and their functionalized forms to treat hazardous compounds that can be found in the composition of post-fire water and wastewater. The most relevant information on the conditions of using the selected materials will be presented, based on a literature review and available patent databases such as Espacenet, GooglePatents, Patentscope, and the European Patent Register (Figure 1). Over the course of the analyzed 10 years (2014–2023), a dynamic development can be observed in the field of topics concerning the application of hydrogels for wastewater treatment. This is evidenced by the continued growth of scientific publications in this area (Figure 1a). In contrast, there are no articles directly related to using hydrogels strictly in post-fire water and wastewater management and a relatively small amount of work regarding the treatment of firewater effluents in general (Figure 1a). On the other hand, given the number of technical solutions protected by patent law (Figure 1b), some activity is observed in the use of hydrogels for wastewater treatment. Nevertheless, no patents related to the treatment/management of post-fire water were found in the searched databases.

Taking into account the lack of comprehensive solutions for the management and pretreatment of wastewater generated during fires, the possibility of using hydrogels and their modification was analyzed in the case of fires in both urbanized and undeveloped areas. Thus, the article will discuss issues related to hydrogels and their functionalization towards the capture, decomposition, and neutralization of selected pollutants, with a particular focus on substances present in fire water/wastewater. In addition, the impact of these materials on treatment efficiency and environmental safety will be analyzed.

## 2. General Characteristics of Hydrogels

Hydrogels are two-phase, hydrophilic materials composed of a three-dimensional polymer network and an aqueous solution that fills the spaces between the cross-linked chains. The hydrophilicity of these materials arises from the presence of chemical groups such as hydroxyl (-OH), carboxyl (-COOH), amide (-CONH-, -CONH_2_), and sulphonic (-SO_3_H), which are located in the main backbone or side chains of the polymer substrate. Hydrogels can also incorporate hydrophobic components by mixing or copolymerizing two types of polymers with different affinities for aqueous solutions. However, in such cases, at least one of the polymers must be hydrophilic [18,19]. Hydrogels can be synthesized from a variety of natural and synthetic polymers or their combinations. Table 2 provides examples of compounds used to create these materials.

The primary advantage of natural polymers lies in their easy biodegradability and high biocompatibility, allowing them to be enzymatically broken down into low-molecular-weight, well-absorbed products. In contrast, synthetic polymers are known for their mechanical strength but degrade much more slowly [21,22]. By combining polymers of different origins, hydrogel materials can be engineered to possess specific properties tailored to particular applications [23]. By controlling factors such as the hydrophilic-hydrophobic ratio, ionization of the polymeric structure, and interactions between individual building blocks, it is possible to influence the degree of cross-linking, porosity, sorption, and transport properties of hydrogels [18,23].

### 2.1. Hydrogel Classification

**Figure 2:**
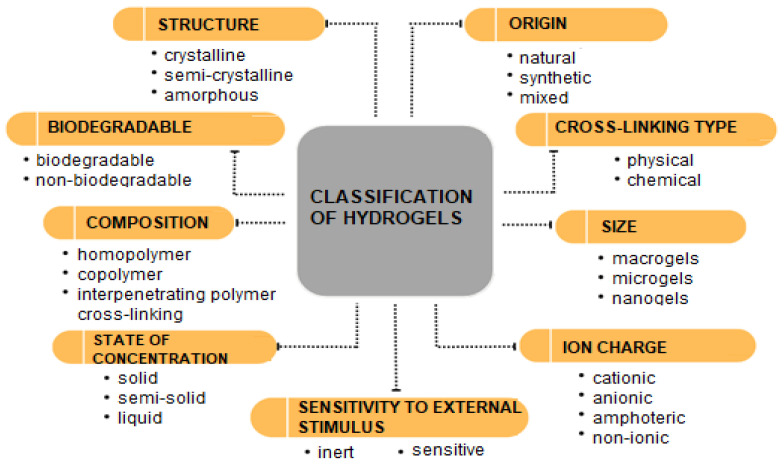
Division of hydrogels based on various classification criteria.

In addition to their classification by origin (natural, synthetic, mixed), hydrogels can also be categorized based on various other criteria such as the type of crosslinking, size, ionic charge, structure, composition, biodegradability, state of aggregation, and sensitivity to external stimuli (Figure 2).

Based on the type of crosslinking, hydrogels can be divided into physical and chemical. In this case, the former are also called reversible hydrogels or pseudo-gels because their three-dimensional polymer network is maintained by non-permanent interchain interactions, e.g., ionic, hydrogen, dipolar, or hydrophobic interactions [24]. These bonds can be destroyed by changes in the physical conditions of the microenvironment, such as ionic strength, pH, or temperature. In contrast, chemical (permanent) hydrogels are those in which the polymer network is maintained by covalent bonds, which ensure the stability of the structure regardless of changes in external factors (e.g., pH, temperature). These materials are chemically stable and do not decompose without complete destruction of macromolecules. Both types of hydrogels exhibit heterogeneous structure, uneven cross-linking, and water distribution [19,22,24,25].

Hydrogels can also be divided by chemical composition. According to this classification, one can distinguish between homopolymers, copolymers, and interpenetrating polymer network (IPN) materials. Homopolymer hydrogels are obtained from a single type of monomer, which is the basic structural unit. On the other hand, copolymers (heteropolymers) are constructs composed of two or more different monomers arranged randomly or as blocks of repeating elements along the chain, at least one of which is hydrophilic [22]. The structure of IPN-type hydrogels is formed by interpenetrating chains derived from two or more polymers of natural and/or synthetic origin [18,26]. Full IPN consists of interpenetrating different polymers that have been cross-linked independently of each other and do not form common chemical bonds. In contrast, in a semi-IPN hydrogel, one polymer is cross-linked and the other is in a linear or branched form, not connected in any way to the first polymer [18,26].

Based on their physical structure, hydrogel materials can be categorized into amorphous (non-crystalline), crystalline, and semi-crystalline types (complexes formed by both amorphous and crystalline phases) [22]. Regarding the charge of the side groups in the polymer chains that constitute the hydrogel structure, these materials can be divided into four types: anionic, cationic, amphoteric, and nonionic [25]. Anionic hydrogels carry negative charges, while cationic ones carry positive charges, both falling under the category of ionic hydrogels. Amphoteric hydrogels can ionize to form both anions and cations, whereas nonionic hydrogels are electrically inert [22]. Hydrogels can also be classified based on the size of the resulting particles into macro- (>100 µm), micro- (0.1–100 µm), and nanogels (<0.1 µm) [27,28].

Considering the state of aggregation of the materials obtained, they can be divided into solid, semi-solid, and liquid [29]. Solid hydrogels are characterized by a permanent, highly cross-linked structure held primarily by covalent bonds. At room temperature, they are solids, but when introduced into water, buffer solutions, or biological fluids, they tend to swell. Thanks to these specific properties, they can be successfully used to produce hydrogels for medical and environmental applications. In contrast, semi-solid hydrogels have a looser network structure based on weaker intermolecular interactions (i.e., van der Waals, hydrogen, electrostatic). They exhibit strong adhesive properties, which is very advantageous for biomedical applications for prolonged dosing of drugs into soft tissues (buccal, sublingual, ocular, vaginal). The last group in this classification is hydrogels, which are liquid at room temperature but at a specific temperature exhibit elasticity and soft tissue-like functional properties. In contrast, when considering the fate of hydrogels in both the human body and the environment, biodegradable or non-biodegradable materials are distinguished. The former are susceptible to biological agents (e.g., hydrolysis in the presence of enzymes or microorganisms) and degrade to harmless, low-molecular-weight end products [23]. Another category by which hydrogels can be classified is sensitivity to external stimuli. In this case, one can distinguish between inert (conventional) and sensitive (smart) materials [24]. Conventional hydrogels include loosely bound hydrophilic polymers, mostly non-ionic, characterized by a significant degree of swelling in water. Stimulus-sensitive materials, on the other hand, change their functional properties (most often the degree of swelling) as a result of their response to a change in various external factors, such as temperature, ionic strength, pH, electric field, magnetic field, ultrasonic radiation, mechanical stress, light, and concentration of selected substances (i.e., glucose, enzymes, antibodies, and antigens) [24,30,31].

### 2.2. Properties of Hydrogels

Specific physicochemical properties that characterize the high application potential of hydrogels include, first of all, the ability to absorb and retain large amounts of aqueous solutions, the possibility of reversible swelling, semi-permeability, and ease of immobilization of a variety of active compounds. Moreover, their functionality also depends on biological properties, such as similarity to natural tissues, biocompatibility, and biodegradability, which characterize hydrogels of natural origin in particular [22,28].

The defining characteristic that sets hydrogels apart from other polymer materials is their remarkable ability to undergo volume changes due to their sorption properties. These cross-linked matrices possess the remarkable capacity to swell by absorbing significant amounts of water—up to 1000 g per gram of dry weight [32]. Among the factors determining the predisposition of hydrogels to absorb aqueous solutions are the characteristic properties of the individual components of the polymer network (e.g., concentration, cross-linking density, nature of functional groups, ionic charge, and hydrophilic-hydrophobic ratio) and the properties of the microenvironment (e.g., pH, ionic strength, or temperature) [33].

Swelling, the process of water absorption by a dry hydrogel, is initiated by the hydration of hydrophilic groups, which are highly polar. As a result, the hydrogel begins to increase in volume, causing the hydrophobic groups to move outward. Then, after all these groups are exposed, osmotic pressure is generated as a result of the effort to equalize saturation conditions around the cross-linked hydrogel structure. This allows additional water to be absorbed through the pores (unoccupied empty spaces between chains) [34]. To determine the degree of swelling, Formula (1) is used:

(1) $$SD\left[\%\right]=\frac{m_{SW}-m_{ID}}{m_{ID}}\times 100\%$$

where: *SD*—degree of swelling [%], *m_ID_*—the initial mass of dry hydrogel (xerogel) before swelling [kg]; *m_SW_*—the mass of wet hydrogel after swelling [kg].

Another parameter describing hydrogel materials is the degree of crosslinking, defined as the average number of crosslinked units (γ) that could possibly be formed in the hydrogel polymer chain as a result of the crosslinking process [35]. This quantity is calculated as the product of the number of functional groups (α) and the average polymer chain size (λ_n_). The polymers that make up the hydrogel are linked by chemical bonds, making it possible to create their three-dimensional network. The formation of such a structure is called crosslinking, during which single covalent bonds, multiple bonds, as well as the most common so-called molecular entanglement can be formed. Crosslinking of polymer chains in the hydrogel structure occurs during polymerization or polycondensation reactions. This leads to the formation of large three-dimensional hydrogel networks that convert absorbed water into gel form. Hydrogel materials with a high degree of cross-linking are more compact and rigid, which is associated with much lower mobility of polymer chains and thus a reduced degree of swelling.

Porosity, or the amount of free space between polymer chains forming a three-dimensional network, is one of the most important parameters determining the structure and properties of hydrogels. The factors determining the porosity of these materials are primarily the type and concentration of the substrates used. Porosity can be determined using Equation (2) [36]:

(2) $$P=\left(1-\frac{\rho }{\rho_{0}}\right)\times 100\%$$

where: *P*—porosity [%]; *ρ*—pore density [kg/m^3^]; *ρ*_0_—total hydrogel density [kg/m^3^].

Porosity, along with the degree of cross-linking, plays a critical role in determining the permeability properties of hydrogel materials. With their favorable swelling ability and the capacity to hold significant amounts of water (a universal solvent for bioactive molecules), hydrogels emerge as efficient matrices for transporting various substances. These may include drugs, enzymes, or growth factors, making them versatile and promising carriers for therapeutic applications [25,33,37].

Another characteristic of hydrogels is elasticity, defined as tensile resistance. This property allows modification of the shape or volume of the material under mechanical influence [38]. Hydrogels, due to their high elasticity, respond to stresses with almost instantaneous and completely reversible deformation. Rapid rearrangement of polymer fragments under stress is possible due to the specific structure of hydrogels, more specifically, their cross-linking and the large number of free spaces between the polymer chains forming this network [38,39].

A favorable property of hydrogels is also their ability to degrade naturally in the environment, that is, to decompose into low-molecular-weight, harmless compounds [40]. In particular, biodegradation processes occurring under the action of microorganisms or enzymes are preferred.

In addition to their use in medicine and pharmaceuticals, the swelling ability of hydrogels and their capacity to hold large amounts of water make them highly beneficial for environmental applications. These properties enable hydrogels to effectively remove contaminants from soil and water and manage waste safely after use [41,42]. It should be noted that within the hydrogel group, there are materials that exhibit rapid changes in their properties in response to even minor stimuli from the external environment. They are then called so-called “smart” polymers [19,43]. Applying the right stimulus causes changes at the micro- and macro-structural levels. At the microstructural level, there can be, among other things, a change in the nature of the surface from hydrophilic to hydrophobic and/or vice versa, or a change in the charge on the particle surface. Macroscopic changes, on the other hand, involve the precipitation of a given substance from solution in the case of linear copolymers, a rapid change in volume in the case of cross-linked copolymers, or a change in the water content bound in the hydrogel structure [19,44].

### 2.3. Functionalization of Hydrogels

Functionalization of materials is nothing more than carrying out changes in their chemical structure or enrichment with various active compounds resulting in the acquisition of new/desired properties by these materials. Also, in the case of hydrogels to improve or obtain entirely new properties dedicated to use in purification and treatment processes for contaminated waters, functionalization can be carried out through these two paths:

- by chemical modification of the polymeric hydrogel network
- by immobilizing a variety of compounds on the surface and/or inside the porous hydrogel matrix.

Chemical modification involves altering the material properties of hydrogels through chemical reactions that introduce new compounds or functional groups into their structure [45,46]. As a result, by appropriately adjusting the components that build the three-dimensional network and creating permanent covalent bonds between the individual components, it is possible to influence the change of physical and chemical properties of these materials. Through rational chemical modification, hydrogels with increased surface hydrophilicity and more favorable sorption properties can be obtained. On the other hand, the second path involving the immobilization of a variety of active molecules in hydrogel matrices is much more commonly used to impart desired functionalities to these materials, enabling, among other things, an increase in processing efficiency and neutralization of harmful compounds contained in aqueous solutions. In this area, the trapping technique is most commonly used [47]. This method can be characterized as a fast, simple, and inexpensive procedure that allows the preservation of all the specific properties of the retained substances. In this case, immobilized molecules are suspended in the free spaces between cross-linked polymer chains, filled with water, buffer, physiological fluid, or other aqueous solutions. Due to this procedure, hydrogels enriched with various additives become functional materials with great practical potential. Nanoparticles, metal oxides, antibodies, drugs and other therapeutics, enzymes, organic compounds, fertilizers and other plant protection compounds, algae, microorganisms, and cells are successfully trapped in hydrogel matrices [48,49,50,51,52,53]. However, when considering the possibility of using hydrogels to effectively monitor and remove contaminants from the environment, these materials are most often enriched with microbial cells, enzymes, and/or active compounds of organic and inorganic origin. Examples of such hydrogel-active ingredient systems are shown in Table 3.

Hydrogels are favorable matrices for the immobilization of microorganisms. These materials enable the retained cells to maintain an adequate lifespan and can be used effectively in wastewater treatment and pollution level monitoring. For this purpose, biodegradable hydrogels (polyvinyl alcohol, sodium alginate, and chitosan) are mostly used. The most commonly immobilized cells are bacteria, but one can also find reports on trapping the fungi, archaeons, yeasts, microalgae, or activated sludge (a blend of various heterotrophic bacteria and protozoa) (Table 3). Hydrogels with immobilized microorganisms have been successfully used in municipal (reduction of BOD, COD, and protein content below recommended levels) [56], industrial (biodegradation of *p*-cresol up to 200 mg/L) [58], or oily wastewater treatment (biodegradation rate approx. 66.5%) [57]. They are used to remove polycyclic aromatic hydrocarbons, nitrogen, heavy metals, phenolic compounds, or petroleum pollutants [54,59]. Another example is the monitoring of water purity; e.g., Arlyapov et al. [55] developed biosensors for determining BOD using bacterial or yeast strains immobilized layer by layer on polyvinyl alcohol-based films. These bioreceptors have high sensitivity and a lower detection limit of BOD of 0.5 mg O_2_/dm^3^ and 0.7 mg O_2_/dm^3^ for the bacterial and yeast biosensors, respectively.

In addition to whole cells, hydrogels are also used as matrices for enzyme immobilization (Table 3). In this way, heterogeneous biocatalyst preparations with increased operational stability, resistance to process conditions, and reusability are obtained [51]. Mostly oxidoreductases catalyzing oxidation and reduction reactions, as well as hydrolases responsible for the hydrolysis of various substances, are subjected to immobilization. The application of these enzymes enables the effective processing of hazardous compounds from effluents such as post-firefighting water. For example, laccase has been successfully used in the neutralization of phenolic derivatives (bisphenol A—degradation of 25–82% in 12 h) [60,73] and polycyclic aromatic hydrocarbons (benzopyrene—degradation of 91% in 96 h) [62].

To increase their suitability for environmental applications, hydrogels can also be functionalized with a variety of organic compounds, such as tannic acid, EDTA, or hexadecylamine (Table 3). As well as with inorganic compounds, where most examples relate to the immobilization of oxides, including iron oxides (FeO, Fe_2_O_3_, γ-Fe_2_O_3_, Fe_3_O_4_), graphene oxide, and alumina (Al_2_O_3_). However, the literature also includes studies on the use of insoluble preparations such as montmorillonite, potassium, nickel hexacyanoferrate, and gold nanoparticles confined within hydrogel matrices (Table 3). It is important to highlight that the development of functionalized hydrogels with enhanced applicability potential represents a significant step forward in the creation of environmentally friendly solutions for the effective capture and purification of wastewater [74].

## 3. Examples of Using Hydrogels to Remove Selected Contaminants Presented in the Composition of Post-Fire Water and Wastewater

Efficient wastewater and water treatment processes are an important issue worldwide. Fire effluents can often be very complex systems, so innovative technologies are needed to reduce and control the release of hazardous substances into the environment. However, their management directions should be focused not only on the neutralization of pollutants but also on the recovery of usable water. Therefore, finding affordable and environmentally friendly solutions to prevent the depletion of available water resources is one of the most important challenges. Nevertheless, the objective should extend beyond the mere reduction of microbiological, chemical, and physical contaminants. It should also encompass the restoration of the treated water to a quality that allows for its reuse, for example, as so-called “grey” water.

**Figure 3:**
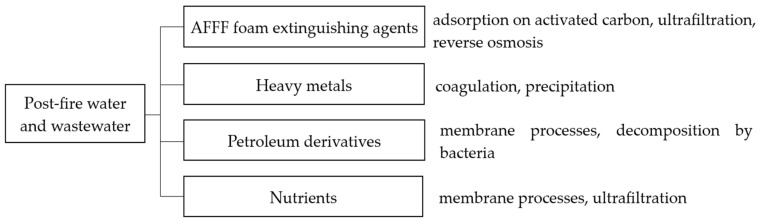
Main components of post-fire water and examples of methods of treating fire wastewater depending on its composition.

Currently, sewage resulting from fires, depending on the place of origin, is treated as hazardous waste or is not subject to management at all. Many treatment plants refuse to accept sewage containing AFFF foam extinguishing agents. Their composition is based on mixtures of hydrocarbons and fluorinated foaming compounds that create an aqueous film, which can significantly affect the sewage treatment process [5,14]. One of the current methods of dealing with fire sewage is to dilute it significantly and introduce it into the stream of industrial or municipal sewage in a way that ensures the volume fraction is sufficiently small to prevent interference with the treatment process. A larger share of fire sewage can be taken into account only if it is previously subjected to appropriate chemical, physicochemical, or microbiological treatment. Fire sewage treatment, mainly aimed at removing AFFF preparations, is carried out by the methods of adsorption on activated carbon, ultrafiltration, and reverse osmosis [75,76,77]. In the case of other pollutants, both processes based on coagulation and precipitation, decomposition by bacteria, and membrane processes are used (Figure 3).

**Figure 4:**
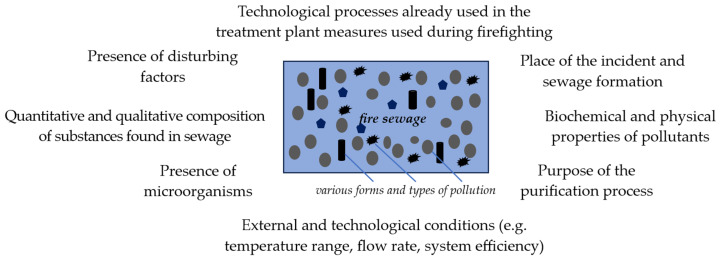
Examples of factors determining the decision on the possibility of using a given technology in the fire sewage treatment process.

However, the selection of a suitable solution for use in wastewater treatment processes requires an analysis that takes into account, among other things, the quantity and quality of substances present in the wastewater or the presence of other interfering agents, such as microorganisms (Figure 4).

Depending on the type of pollutant and the purpose of the work carried out, various solutions are used, including coagulation, sedimentation, flocculation, membrane processes, or biological treatment. One of the most commonly used methods is adsorption, where different types of sorbents are used. Attractive raw materials for the manufacture of sorbents or membranes are hydrogels, which, through functionalization, can be used to remove selected contaminants from water, including drugs, dyes, and heavy metals [78,79,80].

It should be pointed out that materials with the following characteristics are preferred in wastewater treatment: high adsorption capacity, fast removal kinetics, reusability, and cost-effectiveness. Therefore, it is necessary to take into account factors that determine adsorption capacity, including factors such as contact time, pH of the medium, initial adsorbate concentration, mechanical properties, and competition. Optimization of such conditions will determine the maximum adsorption capacity of the hydrogels and allow the development of dedicated, efficient materials [74].

Considering the need to take action in the field of pollutant neutralization present in post-fire water and wastewater capture, as well as water retention in a given area, hydrogels seem to be suitable materials in the case of fire wastewater. Depending on the place of origin, hydrogels can be a good tool in wastewater management during firefighting operations.

### 3.1. Hydrogels in Metal Removal Processes

Metals are substances commonly present in nature. However, the forms of their occurrence are becoming increasingly diverse, and the amount in individual parts of ecosystems is constantly growing as a result of anthropogenic human activity. The occurrence of a fire can additionally lead to the emission of metals in various forms, both as simple and complex compounds, macro- and nano-sized. Their form and concentration are determined by the structure and chemical composition of the elements covered by the fire, as well as the means used in the fire extinguishing process.

An example is forest fires, which can play a significant role in the release of metals usually sequestered in soil organic matter and vegetation and facilitate their movement, increasing the mobility and bioavailability of metals. The results of research conducted in various parts of the world, including the USA, Australia, and Lithuania, indicate the presence of metals such as Cd, Co, Cr, Cu, Hg, Mn, Ni, Pb, Zn, and As in soils and ashes of burnt forests. Surface water sediments [81,82,83,84,85] showed that flame retardants contained V, Cr, Mn, Cu, As, Cd, Sb, Ba, Tl, and Pb in concentrations many times higher (from 4 to 2880 times) than the limits of these metals for drinking water permitted in the USA. Based on the available data, it was also estimated that in the years 2009–2021, the use of flame retardants in the USA contributed to the release of about 380,000 kg of toxic metals into the environment [85]. Therefore, undertaking work on the capture and neutralization of fire water containing metals is an important element in the field of proper water management.

Hydrogels can be used as effective adsorbents for wastewater and contaminated water treatment, separation materials that can be regenerated and reused [79]. In this group, nanocomposite hydrogels, highly porous colloidal structures, are characterized by their high adsorption capacity, which allows them to be used to remove contaminants found in abnormal locations, such as sewers, in the event of fire effluent run-off. Hydrogels with embedded magnetic nanoparticles (MNPs), such as Fe_3_O_4_ nanoparticles, which can be recollected from the sewerage system by applying an external magnetic field, are a special case. The disadvantages of magnetic hydrogels are their brittleness and structural instability when used to fill adsorption columns. Thus, Salahuddin et al. [86] developed gel beads consisting of an alginate polymer acting as a matrix and cellulose nanofibers (NCBF) decorated with MNPs acting as a reinforcing agent. MNPs in the gel increase the adsorption activity and functionality, while CNFs provide good stress transfer when the beads are subjected to compression. The composite gel beads showed high efficiency in the adsorption of simple ions of Al, K, Se, Na, V, and S. According to the authors’ declaration, this adsorbent is safe for users and environmentally friendly [86].

Adsorbent materials of natural origin are of great interest. A promising example of such adsorbents are hydrogels, which form a hydrophilic three-dimensional polymer network that has a high capacity to adsorb large amounts of metal ions and dyes from wastewater. Although hydrogels can also be made from synthetic polymers, natural polymers are much more environmentally friendly. Recently, cellulose-based hydrogels (CBHs) have been intensively studied due to their high availability, biodegradability, non-toxicity, and excellent adsorption capacity. The literature review presented below highlights different CBH adsorbents in the context of the removal of dyes and heavy metals from wastewater using materials produced by different synthesis techniques and using different adsorption mechanisms [87].

Another example is a hydrogel, consisting of polyvinyl alcohol (5–90 parts), chitosan (5–90 parts), sodium carboxymethylcellulose (0–20 parts), graphene oxide (0.05–0.3 parts), and sodium alginate (3–5 parts), which can effectively adsorb heavy metal ions from water. Moreover, it has a high adsorption capacity and selectivity and offers the possibility of cyclic regeneration and recycling. The material is also biodegradable, which is important in terms of environmental impact and the need to reduce waste [88]. A jute-based hydrogel with polyacrylic acid (Jute/PAA) was also developed to remove heavy metals. Adsorption equilibrium for this composite hydrogel was achieved within 10 min for an initial heavy metal ion concentration, especially Cd^2+^ and Pb^2+^, of 40 mg/L and using 1 g/dm^3^ of hydrogel. The material was applied to snow thaw (melting) wastewater to verify the ability to remove divalent ions such as Cd^2+^, Pb^2+^, Cu^2+^, Zn^2+^, and Mn^2+^ and alkali metal ions Mg^2+^, Ca^2+^, K^+^, and Na^+^. Concentrations of Pb^2+^, Cd^2+^, and Cr^2+^ were reduced by 80–90% using an adsorbent of 1 g/dm^3^ over 2 h [89]. Examples of applications of hydrogels and their composites for removing metals from wastewater are included in Table 4.

The adsorption process was found to depend on intramolecular diffusion, chelation, and ionic interactions. The rapid adsorption process in the initial phase occurs at the surface. However, sorption within the hydrogel network and the penetration of metal ions through the pores in the hydrogel matrix slow down the process. Heavy metals such as Cd^2+^, Pb^2+^, Cu^2+^, Zn^2+^, Ni^2+^, and Mn^2+^ can be successfully removed from wastewater using EDTA-functionalized chitosan-polyacrylamide hydrogel with efficiencies ranging from 20% for Mn^2+^ to more than 60% and 80% for Pb^2+^ and Cu^2+^, respectively [66]. On the other hand, a cross-linked matrix based on polyvinyl alcohol, enriched with tragacanth gum functionalized with a copolymer of 2-acrylamido-2-methyl-1-propanesulphonic acid (AMPS) and 1-vinylimidazole (VI), is used for the removal of both heavy metals and industrial dyes with maximum adsorptions of 81.78, 69.67, 94.0, and 101.74 mg/g of hydrogel for Pb^2+^, Cu^2+^, Crystal Violet, and Congo Red, respectively. An interesting solution is using pectin, chitosan, sodium alginate, and three heteropolysaccharides (from maize stalk, ginkgo biloba, and liquorice), which were cross-linked with cellulose from maize stalks, to produce complex hydrogels [96]. The resulting hydrogels were used to efficiently remove Pb^2+^ from aqueous solutions. The ability to adsorb metals was found to be caused by pore-filling effects, electrostatic attraction, and ion-exchange interactions, as well as a large specific surface area (0.11–1.17 m^2^/g) and adequate pore diameter (12.70–33.44 nm) [97]. The efficiency of hydrogel-based aqueous solution purification processes can be impaired by the presence of other substances in the system [87], including, for example, alkali metal salts such as NaCl, MgCl_2_, and CaCl_2_, which determine the chelating capacity of hydrogels. This effect was studied by Shawky et al. [98] in the adsorption of Fe(III), Cu(II), and Mn(II) metal ions by a PVP/AAc hydrogel in the presence of various alkali metal salts [98]. The adsorption of Fe ions was found to be unaffected by NaCl even at high concentrations, while higher concentrations of CaCl_2_ and MgCl_2_, resulted in a slight decrease in adsorption. A decrease in the pH of the solutions was also observed, which is explained by the exchange between the metal ions Mn^+^ and H^+^ of the carboxyl group (COOH) in the hydrogel. Moreover, there was a decrease in bicarbonate concentrations and a decrease in calcium and magnesium concentrations [99].

### 3.2. Hydrogels in Processes of Removing Organic Compounds

An important group of pollutants present in post-fire effluents are organic compounds resulting from accidents or contamination following the extinguishing process, due, for example, to the use of various types of extinguishing agents, such as compounds from the group of perfluorochemicals (PFCs) contained in fire extinguishers. However, the emission of organic pollutants depends on the location of the fire and the means used to extinguish the fire. For example, fires in forested catchments pose a risk of water contamination with dissolved organic matter (DOM) that comes from the fire. It should be noted that the composition and reactivity of DOM after a fire is different from DOM produced in biological degradation processes. Frequent fires change the composition of DOM to components that are less bioavailable and increase the degree of aromaticity, which can have a negative impact on water quality. Additionally, it has been shown that the newer the fire, the more aromatic and humified DOM components are found in the environment [100,101]. Organic compounds produced as a result of fires, such as volatile organic compounds (VOCs), PAHs, dioxins, or furans, are mostly associated with ash [82,102,103,104] and can reach surface waters through deposition or surface runoff, eventually dispersing over wide areas, causing environmental contamination or contamination of water supply infrastructure in fire-affected areas [104]. Organic dyes can be released as a result of warehouse fires or other industrial facilities, including food, clothing, paints, and varnishes. The resulting sewage can then have different colors and clearly indicate environmental pollution. Another group is oil pollution, which often requires different actions in terms of neutralization. However, organic compounds, by virtue of their structure, can be both polar and non-polar in nature, so it is necessary to adapt the hydrogel accordingly towards meeting specific requirements. Depending on the modification and functionalization of the hydrogel, it is possible to carry out sorption, separation/isolation, or degradation processes for the contaminant in question.

An interesting solution is the graphene oxide/polyethyleneimine (GO/PEI) composite hydrogel, which is characterized by increased mechanical strength and a focus on eliminating toxic organic dyes from the aqueous environment [105].

Hydrogels with low adhesion superoleophobicity are the ideal basis for modifying filter materials to achieve effective and antifouling oil/water separation. However, there are still some disadvantages that hinder their practical application, which include expensive raw materials, a complex production process, poor stability, and poor durability. Li et al. [106] developed a durable and super-hydrophilic mesh coated with chitosan-alginate hydrogel (CS-ALG) for effective oil/water separation in hypersaline environments. Due to the integration of superhydrophobic surfaces based on polysaccharides and hierarchical micro/nanostructures, the produced CS-ALG hydrogel-coated mesh exhibits excellent superoleophobicity under water and an anti-oil staining effect. As a result, the mesh can separate different oil and water mixtures with a high separation efficiency of up to >99% [106].

Super-hydrophilic and underwater super-oleophobic hydrogel-coated meshes (CAHM) made of calcium alginate (CA) were developed by Wang et al. [107] CA was introduced to produce both hydrophilic chemical compositions and rough structures on the resulting mesh surfaces. The resulting grids, which had an underwater oil wetting angle (UOCA) of ~154.3° and a small oil slip angle (OSA) of ~7°, could separate different oil/water mixtures with efficiencies of more than 99% and maximum water fluxes of up to 28,108.9 L/m^2^·h. This separation process was spontaneous and driven solely by gravity. Moreover, the meshes prepared in this way still retained high stability in the presence of corrosive organic solvents [107]. Another solution for oil-water separation is a stable, super-hydrophilic, and underwater super-oleophobic Fe composite mesh coated with Prussian blue (PB)/alginate hydrogels through coordination-driven in situ self-organization. The PB/AlHs@Fe composite mesh combines oxidation processes (AOP) with the hydrophilic properties of alginate hydrogels (AH). High self-cleaning efficiency through peroxodisulphate (PMS) and H_2_O_2_ activation is achieved, with a good wetting angle of the subsea oil (OCA > 156°). Thus, the composite mesh was characterized by a high oil-water separation efficiency (separation efficiencies of up to 99.5%), with a water flux of 180,000–280,000 L/m^2^·h. In addition, it has shown exceptional stability, durable alkali resistance, and salt resistance in oil/water separation [108].

Also of interest is the nanofibrillated cellulose (NFC) hydrogel, which was used to modify a hydrated ordinary cellulose filter for oil/water separation [109]. A material with hydrophilic and oleophobic properties, an extended service life, and reduced environmental impact was obtained. An efficiency of ≥99% under gravity was obtained with a water jet exposure of 89.6 L/m^2^·h. The NFC hydrogel forms a hydrated layer when it has reached its maximum swell and provides a large surface energy difference between the oil and the filter. The modified filter prevents oil clogging inside the filter with NFC hydrogels and allows water to flow through while preventing oil ingress [109].

For the reduction of organic pollutants, enzyme-modified hydrogels are used. For example, Bilal et al. [110] developed an agarose-chitosan hydrogel using N-hydroxysuccinimide (NHS) as a mild chemical crosslinking agent. Horseradish peroxidase immobilized on agarose-chitosan hydrogel (ACH-HRP) showed broad working pH and temperature stability, with higher catalytic activity obtained at 50 and 70 °C [110]. One of the most common applications of hydrogels enriched with organic compounds is the removal of various types of industrial dyes. Amongst all the examples given are the use of a cellulose hydrogel enriched with iron(III) oxide (Fe_2_O_3_) for the removal of methyl orange (maximum adsorption equal to 0.033 mmol/g dry hydrogel) [111], a chitosan matrix containing added montmorillonite for the removal of methyl green (maximum adsorption equal to 303.21 mg/g dry hydrogel) [112], or carbon nanotubes for removal of food dyes (maximum adsorption equal to 1508 and 1480 mg/g dry hydrogel for Food Red 17 (FdR17) and Food Blue 1 (FdB1) dyes, respectively) [113], or a hybrid hydrogel based on chitosan and polyvinyl alcohol with immobilized graphene oxide for the removal of Congo Red (maximum adsorption equal to 2704 mg/g dry hydrogel) [114]. Hydrogels also offer the possibility of separating selected compounds, as exemplified by hybrid hydrogels of hyperbranched poly(ether amine)s (hPEAs), allowing the controlled separation of Ponceau S (PS) dye from PS/MB (Methylene Blue trihydrate) dye mixtures [115], and poly(vinyl alcohol) (PVA)-enhanced hybrid hydrogels of hyperbranched poly(ether amine) (hPEAs) for the separation of PS/BY (Ponceau S/Bismarck Brown Y) and OG/MB (Orange G/Methylene Blue trihydrate) dye mixtures [116]. Other examples of hydrogel applications for the removal of selected organic substances that may be present in fire effluents are shown in Table 5.

It is worth noting here that hydrogels modified with bioactive substances or microorganisms allows for not only the separation of hydrophobic substances from aqueous solutions but also the decomposition of organic compounds into simpler compounds. For example, Zhang et al. [73] developed a composite hydrogel consisting of dopamine-functionalized cellulose nanofibers and alginate to immobilize laccase. Under optimal conditions, more than 82% of bisphenol A (BPA) was removed using this heterogeneous biocatalyst. Moreover, the addition of a small amount of 2,2-Azino-bis (3-ethylbenzothiazoline-6-sulphonic acid) (ABTS) resulted in enhanced BPA removal efficiency of 98.7%. In this case, the immobilized enzyme preparation could be easily separated from the reaction system and reused. After 14 cycles of operation, it retained 79.6% of its initial activity [73].

Farid et al. [57], on the other hand, developed PVA-alginate-clay composite hydrogel globules with different types of embedded mineral clays and tested them as a carrier for two marine bacterial isolates, namely *Pseudomonas stutzeri* and *Rhodococcus qingshengii*. The system was evaluated in terms of its ability to decompose crude oil under different environmental conditions, including pH, temperature, and inoculum concentration. The results showed that moderate shaking at 150 rpm and incubation at 30 °C, at neutral pH, created the most favorable conditions for crude oil biodegradation. In addition, the introduction of clay, especially Attapulgite (ATP) mineral clay, into the polymer matrix increased their mechanical and thermal stability, reduced their swelling ability, and improved their biodegradation efficiency [57].

## 4. The Management of Hydrogels During and After Their Utilization in Fire Wastewater Processing

In the process of neutralizing contaminants resulting from firefighting activities or a leak, various types of sorbents are used, including:

- Mineral (made from, among others, calcined or uncalcined diatomaceous earth),
- Organic (based on natural materials such as cellulose or plant fibers),
- Synthetic (PUR foams, polypropylene sorbents, etc.).

According to the requirements, these materials must meet numerous criteria, such as the absorption capacity of the reference hydrocarbon (i.e., diesel oil meeting the requirements of the PN-EN 590 standard [121] for diesel oil grades for temperate climates), grain size, and bulk density value. Sorbents used to collect contaminants from water surfaces are also verifiable in terms of buoyancy, which means that 95% of the sorbent used on water surfaces, both ready-to-use and used, fully saturated with a reference hydrocarbon, must remain on the surface of standing water for 24 h.

As a result of pollutant sorption, waste is generated that requires appropriate disposal, which is one of the main disadvantages of sorbents. These wastes are treated in the legislation of various countries as waste from the oil and gas industry and are therefore subject to disposal as hazardous waste. In the case of some sorbents, recovery is possible by burning the pollutants at high temperatures with an increased share of oxygen. Then, all pollutants are oxidized to carbon dioxide and water [122], and the material obtained in this way can be reused as a sorbent or, for example, as a building material. However, this process is expensive, and for this reason other methods are sought that allow for safe disposal with lower energy inputs.

It should be noted that according to the ASTM F726-12 Standard [123] Test Method for Sorbent Performance of Adsorbents, a sorbent is a substance that can absorb substances absorbed in an amount exceeding 50% of its own weight in relation to both water and petroleum substances. The reference of the sorbent definition to water absorption coincides with the definition of a hydrogel, whereby in the case of hydrogels, the absorption capacity can be up to several hundred percent. Additionally, some materials used for the production of hydrogels, e.g., poly(ethylene glycol), exhibit good properties of both hydrophilic and oleophilic and thus have the potential to work in the field of collecting both sewage and substances insoluble in sewage, such as petroleum substances.

To effectively apply hydrogels in wastewater treatment, they must exhibit essential properties such as swelling ability, high water retention, low production cost, and the capacity to adsorb a wide range of toxic water pollutants. Additionally, they should demonstrate self-healing capabilities to recover from structural damage post-swelling, as well as the potential for reuse and regeneration [22,74]. The use of natural hydrogels in wastewater treatment is highly regarded due to their long-term sustainability, environmental friendliness, biocompatibility, multifunctionality, and high selectivity. Additionally, the stimuli-responsive and smart properties of hydrogels enable a quick response toward water pollution [124]. The functionalization process enables hydrogels to operate effectively across a wide pH range, which allows acting in [125]. The incorporation of nanoparticles (GO, NPs, CNTs, and CQDs) can further enhance hydrogel selectivity toward water pollutants and improve their mechanical properties—one of the main limitations of hydrogel use in wastewater treatment. Furthermore, weakened mechanical strength may occur due to repeated swelling cycles or the nature of the treatment process. Additionally, hydrogels are prone to biofouling, which can reduce their efficiency [126]. Although natural polymers are biodegradable, the loss of mechanical toughness following prolonged swelling in water can render them unrecyclable. The recycling challenge becomes even more complex for synthetic hydrogels, which often require disposal in waste storage facilities [74].

The ability to modify and functionalize hydrogels enables their efficient use in the removal of various streams of water pollutants, as they can retain or reversibly release absorbed substances. Consequently, the applicability of polymeric materials in wastewater treatment processes should be evaluated based on the characteristics of the effluent and the prevailing environmental conditions.

Appropriate management of hydrogels after their use in wastewater treatment is crucial for environmental safety. Such spent materials, particularly those containing hazardous substances, can pose a real contamination risk if improperly disposed of. Therefore, potential environmental impacts and their consequences should be obligatorily considered during the synthesis and application of particular hydrogel-based systems [79]. The main concerns include the toxicity of components, material persistence in the environment, and challenges in regeneration, reusing, and disposal approaches [127]. Regarding toxicity, particular consideration should be given to hydrogel composites, especially those impregnated with heavy metals, organic pollutants, or magnetic nanoparticles, which may leach into the environment during their exposition and after usage. As well, degradation products of synthetic hydrogels, such as polyacrylamides or other polymers, can also contribute to water contamination. In turn, taking into account the storage durability, the huge problem is that many hydrogels are not biodegradable or require a long time to decompose, which could lead to long-term accumulation in landfills and water effluents. Moreover, such disposal of spent materials may pose additional risks of leaching of adsorbed pollutants to soil, water, and living organisms. Another challenge is the complexity of hydrogel compositions, which can make recycling difficult, requiring specialized facilities to handle or neutralize contaminants effectively.

To address these problems, several significant mitigation strategies can be defined:

- Green synthesis approaches, such as the employment of renewable or biodegradable polymers in hydrogel production (e.g., alginate, chitosan, cellulose) or replacing toxic crosslinking or other agents with eco-friendly substitutes [126].
- Life cycle assessment implementation to evaluate the environmental footprint of hydrogels starting from production to disposal state and incorporate design principles to ensure the end-of-life stage involves minimal environmental impact [128].
- Intensified development of hydrogel regeneration and recycling techniques, e.g., by simple desorption processes using low-cost reagents, such as low-concentrated HCl (0.1 M) in the case of heavy metals (Pb^2+^, Cd^2+^) and ethanol for organic pollutants (e.g., p-nitrophenol) [74].
- Increasing the effectivity of recovering valuable components from spent hydrogels, such as adsorbed contaminants or embedded nanoparticles, to minimize hazardous waste storage. As well as using chemical dewatering of hydrogels after sorption of large amounts of pollutants to recover purified water for repeated usage [129].
- Strictly established disposal pathways for a given type of hydrogel matrix, e.g., in the case of biodegradable hydrogels, direct transfer to composting facilities, or for materials based on synthetic polymers, their incineration in controlled conditions to prevent toxic emissions to the environment.
- Encourage the implementation of formal regulations requiring disclosure of hydrogel components and disposal recommendations because current legal regulations lack clear and consistent standards and guidelines for the quality, performance, and safety of adsorbents, including hydrogels, as well as the compliance and monitoring of their application in wastewater treatment [127].

In summary, in the field of processing hydrogels during their application in the removal of pollutants from the water as well as in their post-treatment management, research needs should be taken into account, including systematic studies on the environmental degradation of hydrogels and their by-products and understanding the long-term ecological impact of spent hydrogel residues, particularly composite materials, and bridging the gap between lab-scale green hydrogel production and commercial-scale application.

The costs of producing hydrogels and modified hydrogels are determined by numerous factors, including the size of the company, the technology used in the production process, the modification method, the reagents used, and the sales volume. However, taking into account the growing awareness of society in the field of water resource protection, the use of hydrogels also in the area of post-fire water capture and neutralization of pollutants contained in them seems to be a good direction in water and sewage management.

## 5. Conclusions

The conducted review of scientific literature and patent databases allows a preliminary assessment in the area of the possibility of treating wastewater and post-fire water based on modern solutions such as hydrogels and their modified forms. Numerous developments, modifications, and functionalizations of hydrogels using organic and inorganic substances, nanostructures, or microorganisms and enzymes indicate the possibility of obtaining a material dedicated for the complete management of a given contaminant. Furthermore, it is crucial to consider the environmental impact of hydrogel matrices. One promising approach for reducing this impact is to explore the use of natural components, such as cotton, alginates, or chitosan, in the development of functional hydrogel formulations. In addition, the design of novel smart hydrogels could also prove beneficial in terms of their ability to carry out photocatalytic or solar degradation of pollutants, regeneration capability, or biodegradation after the material is used.

However, it is necessary to specify the conditions under which the hydrogel/hydrogel composite can be used or what factors may interfere with effective treatment. In the case of fire wastewater, these issues are crucial, since often the location of the wastewater determines the feasibility of a particular solution. Materials should allow for both wastewater capture and treatment at the site of the incident, i.e., fire or contamination. Nevertheless, the concentration and variety of pollutants present in the wastewater indicate that a system of solutions is necessary for firefighting wastewater, including materials that allow simultaneous adsorption of metals and organic compounds, as well as degradation of organic substances accumulated in the material to simpler compounds.

Given the intensification of efforts to halt degenerative environmental changes and ensure free access to drinking water worldwide, it is essential to test hydrogels as functional materials in purification and treatment processes for contaminated waters. At the same time, it should be remembered that in order to be used effectively, these materials should have specific properties. It is assumed that for this application, ideal hydrogels should have the characteristics to which they belong [22]:

- Stable three-dimensional structure
- High capacity of absorption
- High rate of fluid absorption in a reversible manner
- High durability and stability during the swelling process and storage conditions
- Good mechanical resistance
- Thermal and chemical resistance
- High flexibility of the polymer structure
- Colorless and odorless
- Photostability
- Complete lack of toxicity
- High biodegradability to low-molecular-weight environmentally friendly compounds
- Possibility of regeneration and reuse
- Depending on the application requirements, the hydrogel must be able to return the absorbed solution or hold it
- Depending on the application, it is possible to enrich the material with active substances (e.g., enzymes that break down different groups of water-soluble contaminants)
- Low cost

Certainly, it is not possible for a given hydrogel to have all the above-mentioned features at the same time. In fact, components that enable the maximum level of some of these functionalities will lead to inefficiencies in others. Therefore, in practice, production variables must be optimized to strike the right balance between desired properties for the target application.

## Figures and Tables

**Table 1:** The range of concentrations of selected compounds in fire effluents and water samples from the Gęśnik river after the landfill fire in 2023, in relation to the annual average (AA) environmental quality standard (EQS) for surface water quality as defined in the EU Directive with its annexes and a class 1 lowland sandy loam river, as required by law in Poland, for [2,8,9,10,11].

Parameter/Relation	Unit	Inland Surface Waters (AA-EQS) [8]	Lowland River, Class 1 [9]	Fire Wastewater [10]	Gęśnik River ^a^ [11]	Gęśnik River ^b^ [11]
pH		-	7.4–8.0	-	7.4 ± 0.3	-
BOD5	mg O_2_/dm^3^	-	≤2.6	1900–11,000	-	-
COD-Cr	mg O_2_/dm^3^	-	≤25	3800–860,000	14.1 ± 3.2	28,000 ± 630
Total suspended solids	mg/dm^3^	-	≤11.0	49–92	-	-
Phenol	µg/dm^3^	-	≤10	99–180	-	-
Mineral oil index	mg/dm^3^	-	≤0.2	55.2	<0.05 (0.05 ± 0.02) *	7.45 ± 2.77
Mercury	µg/dm^3^	0.05	- **	-	<0.020 (0.020 ± 0.005) *	0.47 ± 0.12
Nickel	µg/dm^3^	20	4 **	-	100 ± 30	840 ± 230
Naphthalene	µg/dm^3^	2.4	2	-	<0.001 (0.0010 ± 0.0004) *	1.36 ± 0.12
Antracen	µg/dm^3^	0.1	0.1	-	<0.001 (0.0010 ± 0.0004) *	0.54 ± 0.12
Fluoranthene	µg/dm^3^	0.1	6.3 × 10^−3^	-	0.0027 ± 0.0006	0.71 ± 0.16
Benzo(b)fluoroanten	µg/dm^3^	∑ = 0.03	-	-	0.0015 ± 0.0005	0.45 ± 0.14
Benzo(k)fluoroanten	µg/dm^3^		-	-	0.00068 ± 0.00023	0.19 ± 0.06
Benzo(a)pyrene	µg/dm^3^	0.05	1.7 × 10^−4^	-	0.012 ± 0.002	0.41 ± 0.08
Benzo(g,h,i)-perylene	µg/dm^3^	∑ = 0.002	-	-	0.0012 ± 0.0005	0.24 ± 0.09
Indeno(1,2,3-cd)-pyrene	µg/dm^3^		-	-	0.0019 ± 0.0005	0.25 ± 0.06
Di(2-ethylhexyl)-phthalate	µg/dm^3^	1.3	1.3	465–920	-	-
Benzene	µg/dm^3^	10	10	42.5	-	-

^a^—GIOŚ Zielona Góra, sample number 903/ZG, measurement time: 16.50, Gęśnik river m. Czerwieńsk, ul. Łężycka 28 by the bridge; ^b^—GIOŚ Zielona Góra, sample number 904/ZG, measurement time 14.45, Gęśnik river m. Przylep behind the plot; *—the value of the lower limit of the measurement range of the accredited method, together with the uncertainty; **—AA-EQS for natural watercourses; BOD5—Biological oxygen demand determined after 5 days of incubation; COD-Cr—Chemical oxygen demand determined using dichromate.

**Table 2:** Examples of natural and synthetic polymers used to obtain hydrogel materials [20].

Type of Polymer	Examples of Material		
Natural	Sodium alginate Chitosan Dextran Starch	Pullulan Carrageenan Cellulose Agarose	Polylysine Collagen Gelatin Soy proteins
Synthetic	Polyvinyl alcohol (PVA) Poly(ethylene oxide) (PEG) Poly(propylene oxide) (PPO) Hydroxypropyl methacrylate (HPMA)	Polyacrylamide (PAM) Polyhydroxybutyrate (PHB) Polycaprolactone (PCL) N-isopropylacrylamide (NIPAAm)	

**Table 3:** Examples of solutions used for the functionalization of hydrogels.

Hydrogel	Functionalization	Ref.
**functionalization with microbial cells**		
sodium alginate	*Pseudomonas Monteilii*, *Gordonia* sp.	[54]
polyvinyl alcohol	*Bacillus proteolyticus*, *Pseudomonas yeei*, *Pseudomonas veronii*, *Ogataea angusta*, *Blastobotrys adeninivorans*, *Debaryomyces hansenii*	[55]
	*Bacillus pseudomycoides*	[56]
polyvinyl alcohol, sodium alginate	*Pseudomonas stutzeri*, *Rhodococcus qingshengii*	[57]
	ammonia-oxidizing archaea	[58]
	nitrifying bacteria, microalgae	[59]
**functionalization with enzymes**		
sodium alginate	laccase	[60]
	horseradish peroxidase	[61]
cellulose, sodium alginate	laccase	[62]
chitosan	horseradish peroxidase	[63]
**functionalization with organic compounds**		
chitosan	hexadecylamine	[64]
hydroxyethylcellulose	tannic acid	[65]
polyacrylamide, chitosan	EDTA	[66]
**functionalization with inorganic compound**		
agarose	graphene oxide	[67]
sodium alginate, polyvinyl alcohol	potassium and nickel hexacyanoferrate	[68]
cellulose	Al_2_O_3_, graphene oxide	[69]
chitosan	montmorillonite	[70]
	FeO	[71]
	gold nanoparticles	[72]

**Table 4:** Examples of applications of hydrogels and their composites for removing metals from wastewater.

Hydrogel/Hydrogel Composite	Removed Metals	Comments	Ref.
Carboxymethyl cellulose (CMC) and 2-acrylamido-2-methylpropanesulphonic acid (AMPS)	Mn^2+^, Co^2+^, Cu^2+^, and Fe^3+^	chelating capacity increases with increasing AMPS content in the hydrogel and with increasing solution pH and metal ion concentration; it is chemically stable, min. 5-fold applicability with the same efficiency	[90]
Glucan/chitosan (GL/CS)	Cu^2+^, Co^2+^, Ni^2+^, Pb^2+^, and Cd^2+^	most optimal conditions: temperature 20 °C, pH 7.0, and 0.01 g adsorbent/dm^3^	[91]
Carboxymethylcellulose (CMC)-based thermoresponsive nanocomposite hydrogel	Cu^2+^ and Pb^2+^	selective in relation to Cu^2+^; involves two main sorption mechanisms: adsorption and ion exchange	[92]
Magnetic anionic hydrogel (nFeMAH)	Cu^2+^ and Ni^2+^	pH insensitivity, fast adsorption kinetics, high reusability, and consistency in magnetic separation; magnetic separation efficiency constant at 99%; main mechanism of metal removal based on ion exchange, possibility of metal oxide formation	[93]
poly(AAm-AAc), poly(AAm-AMPS), poly(AAm-AAc-AMPS), and poly(AAm-MEDSA) hydrogels with varying monomer and comonomer concentrations	Fe^3+^, Hg^2+^, and Cr^3+^	removal based on electrostatic attraction forces; metals are retained within the polymer network; improved compressive strength and thermal stability; temperature and pH sensitivity	[94]
Carboxymethylcellulose (CMC)-Fe_3_O_4_ hydrogel	Cd^2+^	the adsorption capacity in seawater is strongly reduced due to ions in solution that reduce the electrostatic attraction between the carboxylate groups of CMC and Cd(II)	[95]
Hybrid nanocomposites of CNF/alginate and MNP–CNF/alginate hydrogel beads	Al^3+^, Se^4+^, Na^+^, K^+^, and V^4+^	possibility to remove also S; pores smaller than 10 nm (MNP-CNF/alginate also pores in the range 30–100 nm); adsorption capacity of gels is better at lower pollutant concentrations	[86]

**Table 5:** Examples of applications of hydrogels and their composites for the removal of organic matter from wastewater.

Hydrogel/Hydrogel Composite	Removed Compounds	Comments	Ref.
β-cyclodextrin–carboxymethylcellulose-based hydrogel	Bisphenol A (BPA)	Globules, swelling capacity in water at the level of 70–200 mL/g; maximum adsorption at 167 µmol/g	[117]
Ethanethiol-cellulose bead hydrogel	metolachlor	Maximum adsorption capacity of globules is 1300 µmol/g; biodegradable; no removal of metachlor in case of fixed bed	[118]
Poly(acrylamide) and methylcellulose (PAAm-MC) hydrogel	pesticide paraquat	removal is strongly influenced by the concentrations of AAm, MC, and paraquat; the highest adsorption capacity (qeq = 14.3 mg/g) was observed for hydrogels synthesized with 6.0% AAm and 0.75% MC swollen in 45.7 mg/L paraquat solution	[119]
Carboxymethyl cellulose hydrogel	4-chlorophenol and 2,4-dichlorophenoxy acetic acid	The adsorption capacity mainly depends on the type of monomer used and the chemical structure of the impurities, the polarity, and the steric effect of the impurities	[120]
Gold nanoparticles (GNPs) embedded in a cross-linked, biocompatible chitosan polymer matrix	pesticide methyl parathion (MP)	Stable up to 200 °C; modification of the hydrogel with gold nanoparticles of at least 0.5% by weight allows sub-2-fold improvement of sorption capacity	[72]
